# Dental Fistulæ

**Published:** 1889-07

**Authors:** 


### Dental Fistulæ.

The recent report by the *Deutsche Monatschrift fur Zahnheilkunde*, December, 1888, of a remarkable case of dental fistula, in which an opening near the nipple of the left breast was found to be caused by disease of the roots of the first left lower molar tooth, furnishes an illustration of the importance of bearing in mind the possibility that disease of the teeth may manifest itself in comparatively remote lesions. One who is sufficiently impressed with this fact may often find a way to cure sinuses or fistulæ which otherwise would baffle treatment.

It ought to be a rule with physicians and surgeons to examine the teeth of their patients whenever there is the slightest reason to believe that they may be a cause of obscure disorders. This rule applies obviously to fistulous openings upon or near the face; and the case cited shows that what seems an almost incredible distance may be traversed by the products of a diseased tooth. It also applies to other disorders, such as neuralgia, and affections of the ear, which are not infrequently dependent upon disease of the teeth. Various specialists have called attention to this matter at different times: but there is still some propriety in directing the attention of general practitioners to it, and in suggesting to them that they may often find an educated dentist a valuable colleague in the management of their patients.

